# Injectable Chitosan-Based Hydrogels for Trans-Cinnamaldehyde Delivery in the Treatment of Diabetic Foot Ulcer Infections

**DOI:** 10.3390/gels9030262

**Published:** 2023-03-22

**Authors:** Henry Chijcheapaza-Flores, Nicolas Tabary, Feng Chai, Mickaël Maton, Jean-Noel Staelens, Frédéric Cazaux, Christel Neut, Bernard Martel, Nicolas Blanchemain, Maria José Garcia-Fernandez

**Affiliations:** 1 Univ. Lille, INSERM, CHU Lille, U1008—Advanced Drug Delivery Systems and Biomaterials, F-59000 Lille, France; 2 Univ. Lille, CNRS, INRAE, Centrale Lille, UMR 8207—UMET—Unité Matériaux et Transformations, F-59000 Lille, France; 3 Univ. Lille, INSERM, CHU Lille, U1286-INFINITE, F-59000 Lille, France

**Keywords:** hydrogels, drug delivery, diabetic foot ulcers, antimicrobial treatment, cyclodextrins, chitosan

## Abstract

Diabetic foot ulcers (DFU) are among the most common complications in diabetic patients and affect 6.8% of people worldwide. Challenges in the management of this disease are decreased blood diffusion, sclerotic tissues, infection, and antibiotic resistance. Hydrogels are now being used as a new treatment option since they can be used for drug delivery and to improve wound healing. This project aims to combine the properties of hydrogels based on chitosan (CHT) and the polymer of β cyclodextrin (PCD) for local delivery of cinnamaldehyde (CN) in diabetic foot ulcers. This work consisted of the development and characterisation of the hydrogel, the evaluation of the CN release kinetics and cell viability (on a MC3T3 pre-osteoblast cell line), and the evaluation of the antimicrobial and antibiofilm activity (*S. aureus* and *P. aeruginosa*). The results demonstrated the successful development of a cytocompatible (ISO 10993-5) injectable hydrogel with antibacterial (99.99% bacterial reduction) and antibiofilm activity. Furthermore, a partial active molecule release and an increase in hydrogel elasticity were observed in the presence of CN. This leads us to hypothesise that a reaction between CHT and CN (a Schiff base) can occur and that CN could act as a physical crosslinker, thus improving the viscoelastic properties of the hydrogel and limiting CN release.

## 1. Introduction

Diabetic foot ulcers (DFU) are a complication of diabetes mellitus that affect around 6.8% of the world population [1]. Infection results in numerous complications such as poor vascularisation and biofilm formation, which can trigger gangrene or lead to death in around 5% of patients in the first month and 42% after the fifth year [2]. First-line treatment consists of debridement, wound dressing, offloading, revascularisation, and intravenous/oral antibiotic therapy [2]. In the last decades, research has been focused on treatment using functionalised wound dressing [3] or drug delivery systems for local treatment [4]. More recently, hydrogels have been deemed a promising strategy since they mimic the extracellular structure and can also be used as a drug delivery system, which are both essential properties to improve wound healing management [5,6]. Furthermore, hydrogels have been shown to preserve the internal moist environment, to improve debridement, and to prevent or impede the spread of infection by delivering local antibacterial treatment [7].

Hydrogels can be formed by physical and chemical interactions. Chemical hydrogels are known for their high mechanical properties and toxicity, whereas physical hydrogels have low mechanical properties but high biocompatibility [8]. Some of the most studied hydrogel-forming natural polymers are chitosan, collagen, and cellulose [9,10,11]. Chitosan (CHT) is a cationic polysaccharide composed of D-glucosamine and N-acetyl glucosamine units. It is obtained by chitin deacetylation (a compound extracted from crustacean shells or mushrooms). As a biocompatible, bioresorbable, and bioactive polymer, it has been widely used for the development of biomaterials [12], such as wound dressings [13], nano- or microparticles [14], and scaffolds [9]. Several combinations have been made to improve the antimicrobial activity of CHT, such as quaternisation [15], the use of drugs such as doxycycline [16], metallic particles [17], and plant-derived extracts [17]. The combination of CHT with other polymers via the formation of polyelectrolyte complexes (PEC) is a promising strategy to improve its intrinsic viscoelastic properties, resulting in physical hydrogels. Anionic polymers used include alginate [14], collagen [18], hyaluronic acid [19], or synthetic polymers [5,20].

On the other hand, cyclodextrins (CD) are a cyclic-form family of oligosaccharides structured by 6, 7, or 8 glucose units joined by α-1,4 glycosidic bonds [21]. CDs have been widely used for the formation of “host-guest” complexes, trapping pharmacological molecules and targeting local treatments. The nature of complex formation is achieved by reversible bonds in the internal cavity of the CD, thus avoiding drug retention [22]. Furthermore, CDs have the property to improve the physicochemical properties, the solubility of non-soluble molecules, bioavailability, and stability [21,22]. Some new approaches to improve CD properties are modification with hydroxypropyl, sulfate, or methyl groups or CD polymerisation like the epichlorohydrin β-Cyclodextrin polymer proposed by Rohner et al. [23] for drug delivery in wound healing or the citric acid polymer of cyclodextrin proposed by Martel et al. [24]. The latter presented poly(cyclodextrin citrate), an anionic carboxylate polymer, and reported successful viscoelastic hydrogel formation with CHT through PEC interactions [25].

Recently, the potential of new drug derivates or plant-derived molecules like phenolic or polyphenolic compounds with high activity against bacteria proliferation and biofilm formation has attracted attention as a novel treatment option. The compounds are characterised by at least one phenolic group in their molecular structure. Some examples are flavonoids, tannins, stilbenes, lignin, and cinnamaldehyde. Cinnamaldehyde (CN) is a natural phenolic compound obtained from the essential oil of cinnamon (about 98% of the total oil volume) with a high activity against Gram (+) and Gram (−) bacteria. The CN mechanism of action has still not been completely clarified, but the most coherent hypothesis proposes that the lipophilic nature of CN enables the disruption of lipid membranes in bacterial cell walls, which can destabilise bacteria and lead to their death [26].

Our research is based on the previous results reported by Flores C et al. [27] and Palomino-Durand et al. [25] on the development of CHT/PCD hydrogels and sponges. Based on all these aspects, the aim of this project was to develop an injectable hydrogel composed of chitosan/citric acid, beta-cyclodextrin polymer (PCD)/cinnamaldehyde (CN) for the treatment of diabetic foot ulcers. The challenge was to incorporate CN in the formation of hydrogel to obtain antibiofilm activity without losing their intrinsic properties (injectability, stability, and hydrogel formation). We first performed a formulation step and then cohesion and stability steps in the physiological medium of each hydrogel and the control. Afterwards, we characterised the samples and studied the activity against bacteria and biofilm eradication.

## 2. Results and Discussion

### 2.1. Cinnamaldehyde—Cyclodextrin Complexation Study

#### 2.1.1. Phase Solubility Diagram

Cyclodextrin’s properties as a host-guest molecule have been widely studied as a means of improving the physicochemical properties of drugs [28]. Poly(cyclodextrin citrate) (PCD) has shown enhanced solubilising properties compared to native CD [27,29]. Table 1 presents the Kf and CE values obtained in the presence of βCD and PCD.

CN has a low intrinsic solubility in aqueous solutions (15.89 ± 0.47 mM). PCD has a Kf constant value of 119 M^−1^ while βCD was only measured at 21 M^−1^. A similar gap was obtained in the CE calculation (0.38 ± 0.1 and 1.67 ± 0.2 for βCD and PCD, respectively). Thus, we can conclude there is significant (*p* < 0.05) PCD activity on the CN solubility. Similar results have previously been observed by Hill L. et al. (2012) [30], who reported a Kf constant value of 28.47 M^−1^ for the complexation with the βCD. Furthermore, Yildiz Z. et al. (2019) [31] did a comparison between the Kf value of CN with hydroxypropyl βCD and hydroxypropyl α CD. Their research demonstrated the higher affinity of βCD cavity for complexation with CN. In fact, Kf values of 140 M^−1^ for HP βCD and 110 M−^1^ for HP αCD were found. Therefore, we can conclude that the complexation of βCD cavity with CN will be higher compared to αCD cavity.

#### 2.1.2. Nuclear Magnetic Resonance (NMR) Study

**Figure 1:**
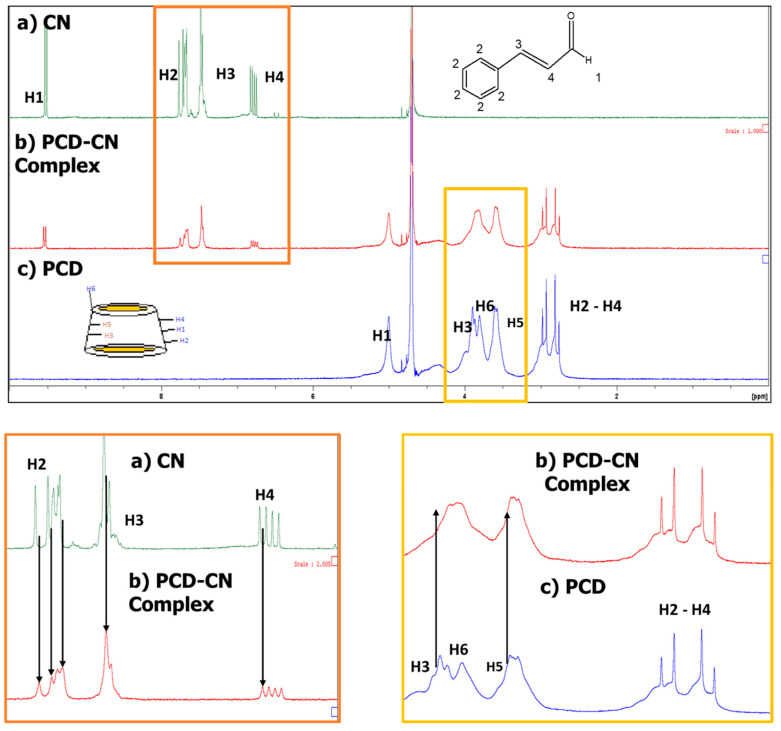
H^1^ NMR spectra of PCD (**c**), PCD:CN complex (**b**), and CN (**a**). A zoom of PCD H3 and H5 proton shifts in the complex spectra compared to PCD spectra is shown in a yellow square. Additionally, a zoom of the H2 (aromatic protons), H3, and H4 shifts from the PCD:CN complex spectra is shown in an orange box.

**Figure 2:**
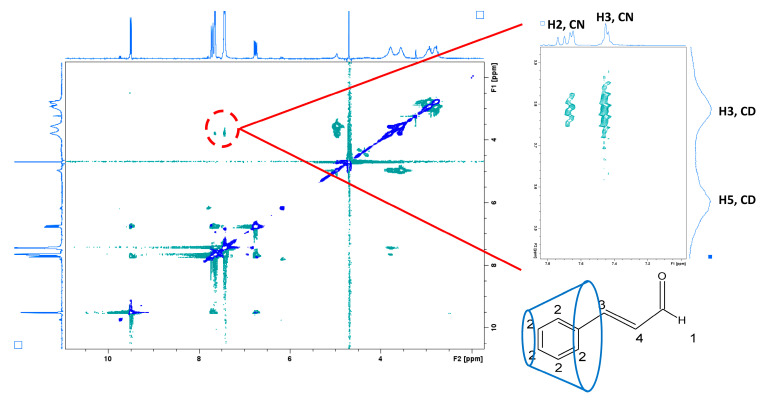
ROESY NMR spectra of the PCD:CN complex (1:1). A zoom focusing on the correlation signals (red circle) of CN (H2 and H3): PCD (H3 and H5) protons are visible beside it. Correlation signals indicate that the aromatic groups (H2) and H3 of CN are complexed by the cavity of CD, as proposed by the model.

The equimolar solution of PCD:CN was compared to a PCD and CN solution in order to identify the proton shift in the spectra of the complex. Results of the ^1^H NMR and ROESY spectra are shown in Figure 1 and Figure 2, respectively. First, the ^1^H NMR spectra corresponding to the PCD show the signal of the glucopyranose unit of cyclodextrins (βCD): H1, H2, H3, H4, and H5 are found at 4.83, 2.93, 3.89, 3.78, and 3.59 ppm, respectively. Concerning the protons corresponding to the CN, the H1, H2, H3, and H4 are found at 9.54, 7.51, 7.45, and 6.79 ppm, respectively [32]. The PCD:CN equimolar solution showed an H3 proton shift in the internal cavity of βCD. Indeed, the H3 proton shifted from 3.89 to 3.85, and no shift for the H5 proton was observed. On the other hand, the CN spectrum shows a shift of the protons corresponding to the aromatic group (H2) and H3 from 7.51 to 7.67 and from 7.45 to 7.49, respectively.

Furthermore, a ROESY NMR study was performed in order to understand the geometries of the inclusion complexes. This test was performed using an equimolar solution of CD cavities in PCD and CN. Figure 2 shows the two-dimensional ROESY spectrum of the complexation. A correlational signal is observed between the H3 and H5 cavities (3–4 ppm) of the cyclodextrin cavities (as in ^1^H NMR) and the aromatic protons of the CN (7–8 ppm region of the CN spectra). Finally, the shift and signal correlation confirm the PCD:CN complexation through the interaction between the CN aromatic group and H3 proton with the internal cavity (H3 proton) of βCD in PCD molecules.

#### 2.1.3. Hydrogel Formation: Vial Turnover Test and Hydrogel Stability

**Figure 3:**
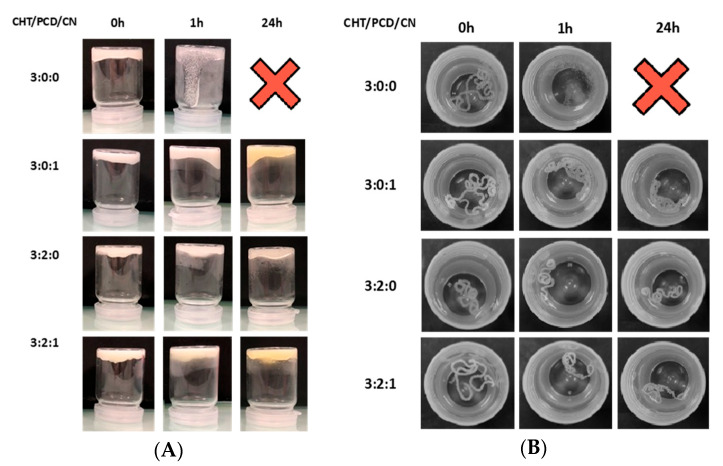
Gel stability tests of controls and CHT/PCD/CN hydrogels immediately after injection (0 h), after 1 h, and after 24 h. (**A**) vial turnover test at 37 °C. (**B**) structural stability of gel cords injected in PBS (pH 7.4) at 37 °C.

Figure 3A shows the results obtained for the vial turnover test of CHT/PCD/CN hydrogel gelation immediately after extrusion of the hydrogel into the vial (0 h) and after incubation of the vial for 1 h and 24 h. The control sample (without PCD and containing only CHT) displayed fast flow from 1 h and complete flow at 24 h. A lower flow rate is observed for the other control containing only CHT and CN (3:0:1), with slight flow at 24 h. In addition, the samples containing PCD (formulations 3:2:0 and 3:2:1) did not display flow within 24 h. Indeed, no visual difference after 24 h was observed.

As shown in the Figure 3B, hydrogels were injected into PBS, in order to evaluate the extruded cord’s stability and cohesion. First, control hydrogel 3:0:0 was dispersed in PBS medium after 1 h, whereas cord made of control 3:0:1 presented better stability and cohesion. Concerning the hydrogel cords 3:2:0 and 3:2:1, both presented good stability after 24 h. Nevertheless, we observed a shrink pattern after 24 h for both samples. This effect is more noticeable in the 3:2:1 formulation.

#### 2.1.4. Rheological Analysis

**Figure 4:**
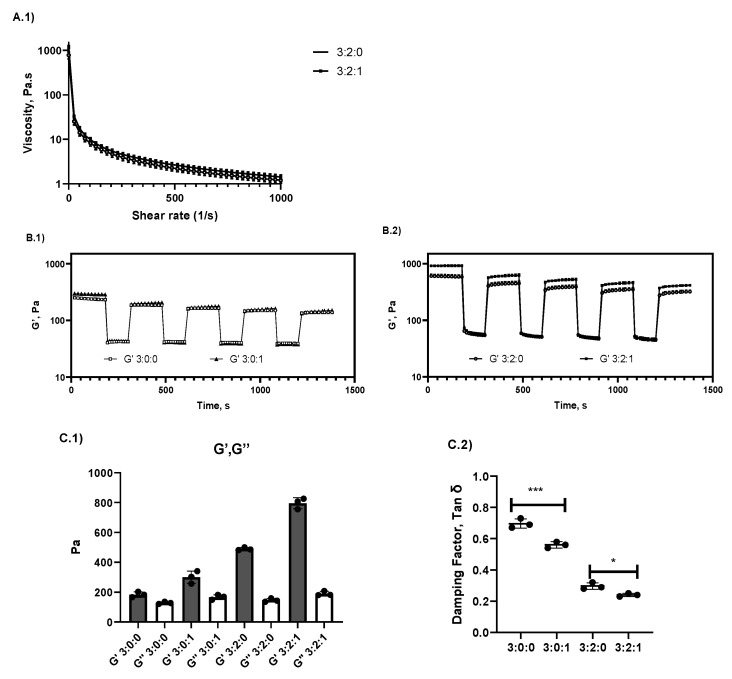
Dynamic viscosity evaluation by increasing the shear rate. The average values obtained (n = 3) CHT/PCD/CN hydrogel formulations 3:2:0 and 3:2:1 are shown (**A.1**). ((**B.1**,**B.2**)) elastic modulus recovery evaluation at 25 °C after a low (1%) and high (500%) shear strain ((**C.1**,**C.2**)) Study of viscoelastic modulus (G′ and G″) and damping factor at 37 °C in the LVR at t = 10 min (*** = *p* < 0.001, * = *p* < 0.05).

A shear rate sweep was performed to study the flow curve since it is an important feature for the development of injectable hydrogel, and the results are shown in Figure 4A. In this context, all samples showed shear-thinning behaviour (viscosity decreases as shear rate increases). Figure 4A shows the viscosity curves obtained for formulations 3:2:0 and 3:2:1 only, since the trends in the curves were comparable with the other two samples. Furthermore, in order to compare the viscosity values obtained at the initial and final shear rates applied, all data values were recorded in Table 2. An increase of initial viscosity from 280 ± 11 to 397 ± 5 Pa.s at a 0.01 shear rate was observed for controls 3:0:0 and 3:0:1, respectively (a 41% increase of viscosity after CN addition). Concerning the 3:2:0 and 3:2:1 formulations, values of 783 ± 72 and 1200 ± 56 Pa.s, respectively, were obtained (53% increase in viscosity after CN addition). Concerning the viscosity values at a high shear rate (1000 s^−1^), values of 1.32 ± 0.07, 0.97 ± 0.22, 1.20 ± 0.16, and 1.42 ± 0.18 Pa.s were obtained for controls 3:0:0 and 3:0:1 and hydrogels 3:2:0 and 3:2:1, respectively. Statistical analysis indicates that CN addition significantly increases (*p* > 0.05) the initial viscosity of the control and hydrogel. However, non-significant differences were found between all samples (*p* < 0.05) at a high rate. Thus, a similar shear-thinning behaviour can be concluded for all samples.

Another important parameter for the development of injectable hydrogels is the recovery of hydrogel elasticity after a high shear strain (also known as the self-healing property, Figure 4B). Samples were tested by applying cycles alternating between a low shear strain of 1% and a high shear strain of 500% in oscillation mode. A substantial decrease of G′ is observed at a high shear strain, and immediate recovery is observed once back to a low shear strain.

Afterwards, hydrogel evaluation of G′ and G″ was assessed at the linear viscoelastic region (LVR) at 37 °C (shear strain of 1% and shear rate of 10 rad/s) in order to differentiate the viscoelastic properties of all the samples (Figure 4C). All samples presented a higher G′ modulus than G″, thus proving the formation of a viscoelastic solid. Despite the low cohesion of formulation 3:0:0, the values of elastic and viscous modulus for this formulation can be explained by the intermolecular hydrogel bonds between acetylated and amino units present in CHT, as previously stated in the literature [25,26]. The damping factor tan δ values were compared, and a significantly higher elasticity was observed for hydrogels once CN was added. A significantly higher elasticity was also observed for the hydrogel 3:2:1 (*p* < 0.05) compared to all samples. A reaction between CHT and CN in the formulation could explain the decrease in the damping factor and the increase in the elastic behaviour of the hydrogel. As a matter of fact, CN could improve the viscoelastic properties by the formation of a Schiff base, as suggested by Rieger, K.A. and Schiffman, J.D. (2014) [33]. Recently, Zhou et al. [34] reported that this type of amphiphilic polymer obtained from Schiff base formation between CHT and CN is able to self-assemble to form nanoparticles.

Therefore, we hypothesise that part of the CN in the hydrogel is linked to chitosan by imine bonds (Schiff base). The phenylpropylene groups of the covalently reacted CN–CHT could interact together through π–π stacking, provoking the self-assembly of the chitosan chains in addition to the polyelectrolyte complex interactions between PCD and CHT (34). In addition, phenylpropylene groups linked to CHT could also interact with CD cavities of PCD by the formation of inclusion complexes (as presented in Section 2.1.2). Thus, the involvement of phenylpropylene groups linked in both types of interactions mentioned above could increase the elasticity of the hydrogel.

Furthermore, in order to evaluate the ratio of bound/free CN in our hydrogels, we calculated the molar ratio of amino groups versus cinnamaldehyde in the starting solid powder formulations (Table 3).

The concentrations of reactive groups of CHT and CN are shown in Table 3. The concentration of amino groups in the formulation is much higher compared to CN, which increases the probability of the formation of a covalent bond, CHT-CN. Further studies need to be undertaken in order to understand the interaction between CHT and CN in the presence of a CD.

#### 2.1.5. CN Release Study and Modelling

**Figure 5:**
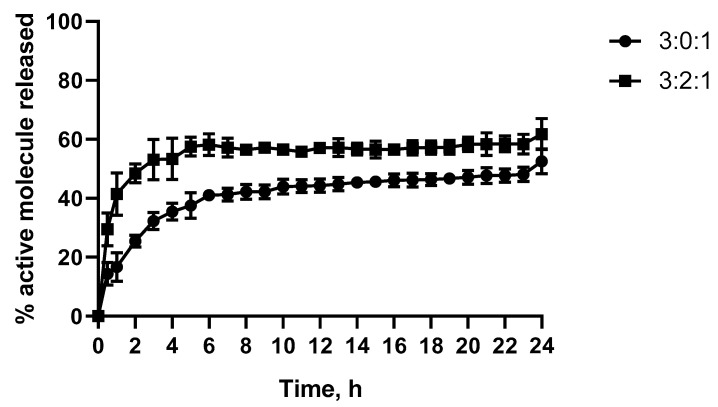
Kinetic release of CN (expressed in %) under flowing conditions in a USP apparatus four dissolution tester (PBS, pH 7.4 at 37 °C).

A release study was performed under dynamic conditions and in physiological conditions (n = 3, PBS pH 7.4 at 37 °C) in order to evaluate the release kinetics of CN and the impact of PCD. Figure 5 shows the percentage of CN released. A partial drug release is observed for both samples. The release kinetics were characterised by a burst release in the first 8 h and a maximal but partial release after 24 h (release of 61% ± 4 and 52% ± 3 of active molecule release for hydrogel 3:2:1 and control 3:0:1, respectively). The percentage of release was compared, and a significantly higher release for hydrogel 3:2:1 was observed (*p* < 0.05). Therefore, in order to explain the increase in CN availability in solutions, a calculation of the concentration of βCD cavity available per gramme of hydrogel was undertaken. A 10 µmol of βCD/g of hydrogel (corresponding to 57% of βCD in PCD as described in Section 4.1) in the formulation was obtained, which explains the 9% increase in active molecule availability in solution (formation of complex 1:1 CN:CD).

The release kinetics data were subsequently fitted into four mechanism models: Zero Order, First Order, Higuchi, and Korsmeyer-Peppas. Linear equation values obtained for both formulations are shown in Table 4. As evidenced, data modelling fitted the model proposed by Korsmeyer-Peppas for polymeric systems (r2 values of 0.96 ± 0.02 and 0.95 ± 0.01 for formulations 3:0:1 and 3:2:1, respectively). As previously mentioned, several drug diffusion mechanisms can be found in a drug delivery system. Based on the results obtained, the concentration gradient (Fickian diffusion) is the main drug transport mechanism involved in the release mechanism of CN from the 3:2:1 hydrogel since the n value obtained is below 0.45 (n = 0.29 ± 0.06). On the other hand, an n value of 0.50 ± 0.02 for control 3:0:1 describes a non-Fickian or anomalous drug transport (0.45 < n < 1). The anomalous transport involves the polymeric chain relaxation (or swelling) in the release profile mechanism. This behaviour can be explained by the reaction between the CHT and CN and the high swelling property of the CHT. Additionally, release constant (k_kp_) values (also described as the drug release velocity) of 18.5 ± 4 and 38.0 ± 6 were obtained for the 3:0:1 and 3:2:1 hydrogels, respectively, which explains the faster release of hydrogel 3:2:1.

#### 2.1.6. Cytotoxicity Test

**Figure 6:**
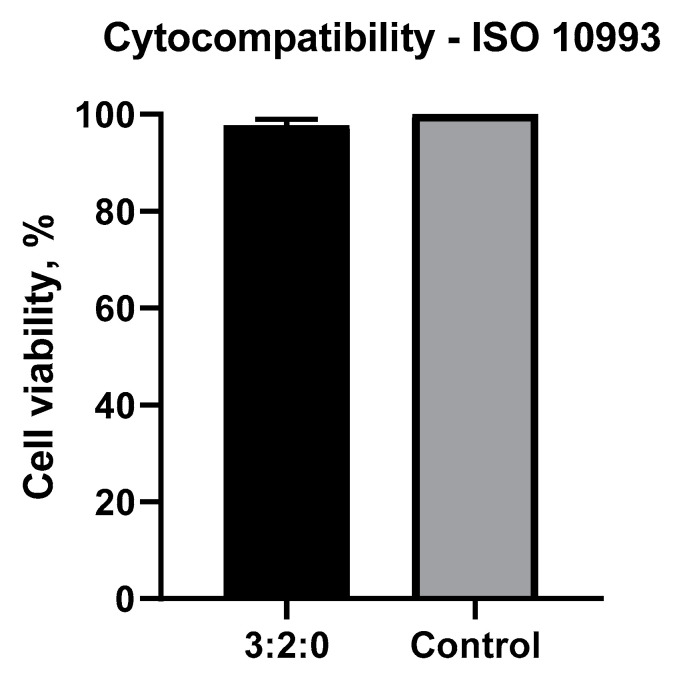
Percentage of cell survival tested on a MC3T3 cell line exposed to an extraction medium from CHT/PCD/CN hydrogels.

Finally, the cytotoxicity of hydrogels was assessed using an extraction method. The formulation without CN was tested and proved the formation of a cytocompatible hydrogel. A pre-osteoblast cell line was selected as it is a clinically relevant cell type involved in diabetic foot osteomyelitis. According to ISO 10993-5, a sample with a minimal cell survival rate of 70% can be considered a cytocompatible sample. In this respect, 99.9% of cell viability observed after incubation over 24 h showed excellent cytocompatibility of the hydrogel (Figure 6).

#### 2.1.7. Antimicrobial Assessment: Kill Time

**Figure 7:**
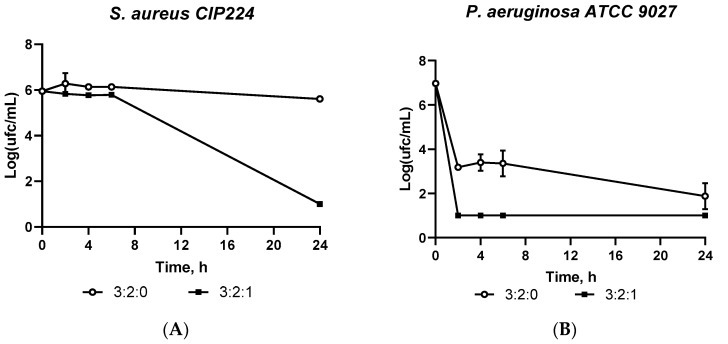
Antibiofilm evaluation in a hydroxyapatite-coated peg over time for (**A**) *S. aureus* and (**B**) *P. aeruginosa*. Bacterial biofilm absorbance after exposition to an extraction medium of CHT/CD/CN hydrogels was measured by OD at 590 nm.

The bacterial kinetic reduction was evaluated to determine the bacterial concentration reduction over time. Samples of formulation 3:2:0 as control and formulation 3:2:1 were studied (n = 3). As observed in Figure 7, no antibacterial activity was observed against *S. aureus* for the hydrogel 3:2:0 whereas formulation 3:2:1 significantly reduced (5 log_10_ reduction) the bacterial concentration between 6 h and 24 h (*p* < 0.05).

On the other hand, a similar and intrinsic activity is observed against a Gram (−) strain. *P. aeruginosa* is a common standard with a high resistance to antimicrobial drug activity. The study carried out on the *P. aeruginosa* strain confirmed the activity of the 3:2:1 hydrogel from the second hour and the intrinsic activity of the 3:2:0 hydrogel (6 log_10_ reduction and 5 log_10_ reduction after 24 h, respectively). This activity has previously been reported by several authors, who proposed that the ammonium group of CHT interacts with the anionic charge of the bacterial cell membrane, therefore, altering the membrane properties (permeability and osmosis) and leading to cell death [35]. Furthermore, some authors have attributed the high bactericidal property of CN to its affinity for the Ftsz protein. Indeed, the Ftsz protein is a 40 KDa protein responsible for the formation of the bacterial cell cytoskeleton in binary fission. CN has an affinity to react with the Ftsz protein by a Schiff base and Michael reactions with amino acids [36].

The results obtained prove the intrinsic activity of hydrogel 3:2:0 and the improvement of the antimicrobial activity of hydrogel 3:2:1. Lastly, a significant difference can be concluded between both formulations after comparing the results using a Student’s *t*-test (*p* < 0.05).

To conclude, these tests confirmed the significant antimicrobial activity of formulation 3:2:1 by inducing a 99.99% bacterial reduction after 24 h for both strains.

#### 2.1.8. Antibiofilm Activity

The antibiofilm evaluation was performed on the same strains used in the previous test (*S. aureus* CIP224 and *P. aeruginosa* ATCC 9027). Formulations of 3:2:1 and 3:2:0 were tested (n = 5), and a brain-heart (BH) broth was used as a control in order to conserve the biofilm structure formed on a hydroxyapatite-coated surface. Samples were compared statistically by using the ANOVA test and Dunnett’s tests.

**Figure 8:**
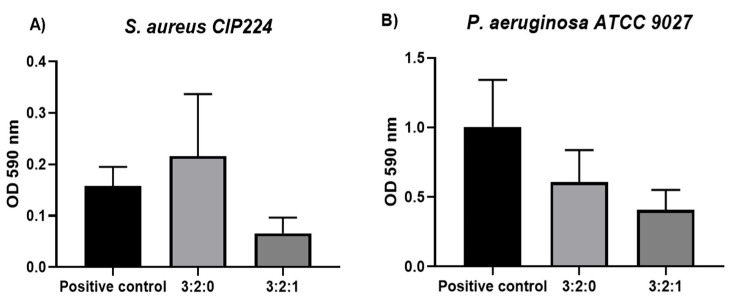
Antibiofilm evaluation in a hydroxyapatite-coated peg over time for (**A**) *S. aureus* and (**B**) *P. aeruginosa*. The bacterial biofilm absorbance after exposure to an extraction medium of CHT/CD/CN hydrogels was measured by OD at 590 nm.

Figure 8 presents the OD values obtained for the samples and the control. On the positive control, the biofilm formation was higher for a *P. aeruginosa* strain than for an *S. aureus* strain, which is consistent with references found in the literature [37]. Indeed, *P. aeruginosa* is the principal species found in complicated DFU infections and the principal cause of DFU aggravation due to its quick proliferation [37]. The activity on an *S. aureus* strain showed non-intrinsic activity of the 3:2:0 hydrogel and significant antibiofilm activity of formulation 3:2:1 (*p* < 0.05). Indeed, a 58% ± 18 bacterial reduction was observed after 24 h. Concerning the *P. aeruginosa* strain, comparable antibiofilm activity (*p* < 0.05) of the hydrogel 3:2:1 (60% ± 14 bacterial reduction) and slight but not significant (*p* > 0.05) intrinsic activity of the formulation 3:2:0 was observed.

The results confirmed the antimicrobial activity of CN against bacteria. Even when the sample is not tested by direct contact with biofilm, intrinsic activity for sample 3:2:0 is observed. This activity can be explained by the dissolution of CHT particles in the extraction medium, which has an antimicrobial activity as already shown in the kill-time test. Some other authors have already reported the enhancement of CHT antimicrobial properties in the presence of CN. Wang X et al. [38] attempted to develop cinnamaldehyde-loaded liposomes decorated with chitosan. This study demonstrated a synergic activity in bacterial wall destabilisation and leakage of the internal cell components in the presence of CHT and CN.

## 3. Conclusions

Injectable hydrogels are an innovative and promising approach for treating DFU. They can be used not only as a drug carrier but also to achieve a sustained release and improve treatments. This study presented a new approach for the use of a naturally derived antimicrobial in a hydrogel by merging the properties of CHT/PCD hydrogel and the antimicrobial properties of CN. Indeed, we have demonstrated the intrinsic properties of a CHT/PCD hydrogel and the improvement of a hydrogel containing CN. Furthermore, an interaction between CHT-CN has been evidenced, which leads us to the hypothesis that CN can act as a “cross-linker” and improve the viscoelastic and antimicrobial properties of CHT hydrogels. The results obtained in this research are the first study for the development of CHT/PCD hydrogels with natural phenolic compounds. Further studies in order to study the interaction of CHT and CN and to achieve the optimal controlled release will be performed in a future study.

## 4. Materials and Methods

### 4.1. Materials

CHT (batch STBJ0437, Sigma Aldrich, Saint-Quentin-Fallavier, France) has a molecular weight (MW) of 256 kD (determined by size exclusion chromatography (SEC)) and a desacetylation degree (DD) of 76% (determined by ^1^H Nuclear Magnetic Resonance (NMR, Bruker, MA, USA)). Poly(cyclodextrin citrate) (PCD) was prepared as described by Martel et al. [30]. Briefly, this method consists of polyesterification between β cyclodextrin (βCD) and citric acid (CTR, Saint-Quentin-Fallavier, France) in the presence of sodium hypophosphite (NaH_2_PO_4_). βCD was provided by Roquette (Kleptose, Lestrem, France). CTR was used as a cross-linking agent, and sodium hypophosphite (NaH_2_PO_4_, Saint-Quentin Fallavier, France) was used as a catalyst. After the reaction, the water was removed in a Rotavapor (Büchi, Flawil, Switzerland), and the solid mixture was treated at 140 °C for 90 min under vacuum. The mixture was then dispersed in water and filtered using a sintered glass funnel. The insoluble fraction (PCDi) was finally obtained after drying at 90 °C overnight, and the soluble fraction (PCD) in the filtrate was further concentrated, purified by dialysis (Spectra/Por^®^, MWCO 20 kDa, Sigma Aldrich, Lesquin, France), and freeze-dried (Alpha 1–2 LDplus, Christ, Germany) at 0.06 mbar and −53 °C. The molar mass of the PCD used in this study was 21 kDa, determined by size exclusion chromatography (SEC) using multiangle light scattering and a differential refractometer. The percentage of cyclodextrin weight was determined at 57% by ^1^H NMR. Lastly, the CN used in this study was obtained from Alfa Aesar (Kandel, Germany) at a purity of 98%.

### 4.2. Methods

#### 4.2.1. Cinnamaldehyde—Cyclodextrin Complexation Study

##### Phase Solubility Diagram

A phase solubility diagram was carried out according to the method first proposed by Higuchi and Connors (1965) [39]. This method has also been used for the study of complexes with drugs or essential oils [21,40,41]. Vials containing 5 mL of an aqueous solution (ultrapure water) of βCD (from 2 mM to 15 mM) and PCD (from 2 mM to 40 mM) were prepared, and an excess amount of CN was added in order to have a final concentration of 151 mM (concentration above CN intrinsic solubility). Then, the solutions were incubated at room temperature for 24 h with constant stirring at 320 rpm. CN solubility was quantified from supernatants by UV-visible spectroscopy (UV-1800 spectrophotometer, Shimadzu, Columbia, Portland, OR, USA) at 285 nm. All samples were prepared in triplicate.

The formation constant (*Kf*) and complexation efficiency (*CE*) were calculated from the slope of phase solubility diagrams in the initial linear range for CN/βCD and CN/PCD complexes. The equation used is as follows:

*Kf* = Slope/*S*_0_ (1 − Slope) (1)

*CE* = Slope/1 − Slope (2)

where *S*_0_ is the intrinsic solubility of CN after 24 h under stirring.

##### Nuclear Magnetic Resonance (NMR) Study

The complex of PCD/CN was studied by ^1^H NMR, and two-dimensional ROESY was performed to characterise the geometry of the interaction between CN and PCD. ^1^H NMR was performed using a solution of CN and PCD in deuterated water (D_2_O), and an equimolar mixture of 10 mM of CN and 10 mM of CD in PCD (calculated from the % of CD in the PCD) was prepared to evaluate the complexation. ROESY was performed using a higher concentration of CN and PCD (25 mM:25 mM) in a deuterated methanol:deuterated water solution (MeOD:D_2_O 1:9). The solution was stirred for 24 h at 37 °C.

First, ^1^H NMR was tested in an AVANCE III 300 MHz spectrometer (Bruker, Billerica, MA, USA) with 16 scans. The ROESY study was performed on an AVANCE III 400 MHz spectrometer (Bruker, Billerica, MA, USA) in order to study the geometry of complexation. Data was acquired in the phase-sensitive mode, and the probe temperature was regulated to 300 K. The data obtained was processed by TopSpin software version 4.0.6.

##### Mixed CHT/PCD Powder Preparation

The first step consisted of grinding CHT and PCD in order to obtain a particle size of less than 125 µM. PCD was milled in a mortar and pestle and then sieved using a 125 µm sieve. The CHT powder was milled using a Fritsch Pulverisette 14 (Idar-Oberstein, Germany) and sieved as previously performed for PCD. Finally, both polymers were co-milled using a Mixer Mill MM400 (Restch, Steinbach, Germany) at a frequency of 10 Hz for 3 min.

##### Hydrogel Preparation

The formulation was prepared and compared to two controls to assess the impact of each compound on hydrogel formation and viscoelastic properties (Table 5). Two syringes connected to each other through a female-female Luer lock connector (Vygon, Ecouen, France) were used to prepare the hydrogel. The CHT/PCD powder was collected in one syringe, and ultrapure water (W) and cinnamaldehyde (CN) were added to the other. Both syringes were connected, and both plungers were pulled alternately for one minute to mix the contents of both syringes. Lactic acid (LA) (volume adjusted for a final concentration of 1% w/w) was then introduced in one syringe, and the mixing step was repeated for a further minute. Finally, a viscoelastic injectable hydrogel was formed in the syringe [42].

**Figure 9:**
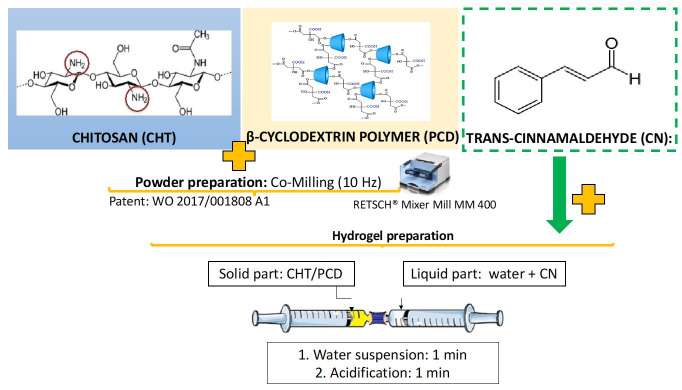
Graphical description of the hydrogel preparation system composed of two syringes interconnected by a female-female Luer lock.

The hydrogel compositions are shown in Table 5, and the hydrogel preparation graphic description is presented in Figure 9.

##### Hydrogel Formation: Vial Turnover Test and Hydrogel Injection in Phosphate-Buffered Saline (PBS)

To evaluate the hydrogel formation, 1 mL of hydrogel was injected into an empty vial (diameter: 2 cm × height: 3 cm). This test is based on the flow resistance property of hydrogels since they have a yield stress, whereas a viscous liquid flows quickly [22]. Based on this, the recipients were inversed and stored at 37 °C. The hydrogel morphology and structural stability at a physiological pH (7.4) were evaluated by injection into PBS at 37 °C. Both tests were visually evaluated after 1 h and 24 h (n = 1).

##### Rheological Analysis

An MCR 301 rheometer (Anton Paar, Les Ulis, France) with a parallel plate geometry of 25 mm (PP25) was used to characterise the viscoelastic properties of hydrogels. A first study of the amplitude and frequency sweep was performed previously to determine the linear viscoelastic range (LVR). Then, the dynamic viscosity (η) of hydrogels was studied in a rotational mode by applying a shear rate from 0 to 1000 s^−1^. Afterwards, the elasticity recovery of hydrogels was evaluated by applying a high and low shear strain of 1% and 500%, respectively, in oscillatory mode. Finally, the hydrogel formation at 37 °C was assessed by measuring the storage and loss moduli (G′ and G″, respectively) in the linear viscoelastic range (LVR).

##### CN Release Kinetics Study and Modelling

CN release was performed using a flow-through dissolution method using a SOTAX^®^ CE7 Smart—USP IV (SOTAX^®^, Aesch, Switzerland). A quantity of 0.2 g of hydrogel was injected directly into each cell container. PBS was used as a dissolution medium (pH 7.4 at 37 °C) at a flow velocity of 5 mL/min. The dissolution medium was coupled to an automatic sampler, Sotax^®^ C615 (SOTAX^®^, Aesch, Switzerland), and a sample volume of 1 mL was taken at each hour up to 24 h. Finally, CN release was quantified using a Nexera Ultra performance liquid chromatograph (LC2040C, Shimadzu^®^, Noisiel, France) at 285 nm by using a C18 reverse phased column (Gemini^®^, 5 µm NX-C18, 250 × 4.6 mm) and a mobile phase composed of acetonitrile/methanol/acetic acid (50/20/30) at a flow rate of 1 mL/min.

Subsequently, four release models were used to compare and understand the drug release mechanism:

Zero-order release: where Q is the amount of drug released or dissolved, Q_0_ is the initial amount of drug in solution, and K_0_ is the zero-order release constant [6]:

Q = Q_0_ + K_0_ t (3)

First-order release: where K is the first-order rate constant expressed in units of time −1. C0 is the initial concentration of drug, and Ct is the concentration of drug in solution at time t. This equation can be expressed as [6]:

dC/dt = −Kt(4)

Higuchi model: where Q is the amount of drug released in time t per unit area A, C is the drug initial concentration, Cs is the drug solubility in the medium, and D is the diffusivity of the drug molecules (diffusion coefficient) in the matrix [43]:

Q = A [D (2C − Cs) Cs t] 1/2(5)

Korsmeyer-Peppas model: where Mt is the cumulative amount of drug released at time t and Mα is the amount of drug released after infinite time. K_KP_ is the constant incorporating structural and geometric characteristics of the drug delivery system [42]:

Mt/M_∞_ = k_KP_.tn (6)

According to the Korsmeyer-Peppas model, exponent “n” can describe four mechanisms of molecule transport in a polymeric system: Fickian diffusion (n < 0.45), anomalous transport (0.45–0.85), case II transport (0.89), and super case II transport (>1).

##### Cytotoxicity Assay

The cytotoxicity of formulation 3:2:0 (hydrogel without CN) was evaluated by the extraction method (indirect contact) according to the ISO 10993-5 standard on a pre-osteoblast MC3T3-E1 cell line (ATCC^®^ CRL-2594™, Manassas, VA, USA). The hydrogels were preconditioned in the Minimum Essential Medium (MEM–α, Gibco^®^, Thermo Fisher Scientific, Illkirch-Graffenstaden, France) at 37 °C and 80 rpm for 2 h. Then, MEM–α was removed, and a concentration of 200 mg of hydrogel/1 mL MEM–α was added. MC3T3–E1 cells were seeded at 4 × 10^3^ cells/well in a 96-well tissue culture polystyrene plate containing 100 µL/well MEM–α medium supplemented with 10% foetal bovine serum (FBS; Gibco^®^, Thermo Fisher Scientific, Illkirch-Graffenstaden, France) and incubated at 37 °C and 5% CO_2_ for 24 h. After incubation, each extraction medium (n = 3) was sterilised using a 0.22 μm filter. Then, the culture medium on the cell layer was replaced by 100 μL of the sterile extraction medium. The cells were incubated for an additional 24 h at 37 °C in an atmosphere of 5% CO_2_. Finally, cell viability was evaluated by fluorometry with the AlamarBlue^®^ reagent (Uptima, Interchim, France). The fluorescence reading was measured at 530 nm as an excitation wavelength and 590 nm as an emission wavelength by a fluorometer (CLARIOstar^®^, BMG Labtech, Ortenberg, Germany).

##### Antimicrobial Assessment: Kill Time

The kill-time test was studied to assess the bacterial reduction over time against *Staphylococcus aureus* CIP224 and *Pseudomonas aeruginosa* ATCC 9027. One mL of hydrogel was injected into a tube containing 9 mL of around 1 × 10^7^ CFU/mL of bacteria suspension in cysteinated Ringer solution (CR). The tubes were incubated at 37 °C, and samples were taken after 2 h, 4 h, 6 h, and 24 h. At each interval, 100 µL were seeded directly on a Mueller Hinton Agar (MHA), and the others with 100 µL were successively diluted 1/10 up to 10^−5^ in a CR solution and seeded as for the non-diluted sample. Finally, the number of viable bacteria were counted after incubation at 37 °C for 24 h and the results were expressed in log CFU/mL. All tests have been performed in triplicate (n = 3)

##### Antibiofilm Study

The same strains as in the previous tests were used at a concentration of about 1 × 10^5^ CFU/mL in brain-heart (BH) broth. An Innovotech^®^ biofilm (Edmonton, AB, Canada) 96-well plate with a hydroxyapatite-coated peg cover was used as a support for the fixation of bacteria biofilm. Therefore, 200 µL of bacterial suspension was added in each well to sink the hydroxyapatite pegs for 48 h at a stirring speed of 60 rpm and at 37 °C. Afterwards, the hydroxyapatite-coated cover was rinsed twice with PBS (pH 7.4) for 3 min each time in order to eliminate the excess bacterial suspension on the peg. Meanwhile, an extraction medium was prepared by sinking 1 mL of hydrogel in 5 mL of BH broth for 24 h at 37 °C. The extraction medium was filtered through a 0.2 µm sterile syringe filter, and the previously prepared biofilm pegs were immersed in 200 µL of extraction medium in a 96-well plate. An incubation of 24 h under stirring was performed, and finally, bacterial biofilm was recovered in PBS under sonication (20 min), and turbidity was quantified by its optical density (OD) at 590 nm. The test was performed in quintuplicate (n = 5).

##### Statistical Analysis

The statistical analysis of the data was performed using the ANOVA test and the Student’s *t*-test. A significance value of 0.05 was considered in order to evaluate the difference between all the samples.

## Figures and Tables

**Table 1:** Values of slope, binding constant (Kf), and complexation efficacy (CE) in the presence of CD and PCD.

	Slope	S_0_ (M)	Kf (M^−1^)	CE
βCD	0.27 ± 0.05	0.016	24.07 ± 6	0.38 ± 0.1
PCD	0.62 ± 0.02	0.016	104.25 ± 12	1.67 ± 0.2

**Table 2:** Mean values of viscosity at a low (0.01 s^−1^) and high shear rate (1000 s^−1^) and mean values of viscoelastic modulus obtained at 37 °C after 10 min.

Formulation	Viscosity (s^−1^)		Viscoelastic Modulus at 10 min	
	0.01	1000	Storage Moduli (G′)	Loss Modulus (G″)
3:0:0	280	0.4	184	128
3:0:1	397	1.0	300	167
3:2:0	783	1.2	489	145
3:2:1	1200	1.4	796	190

**Table 3:** Concentration of reactive groups (amino and aldehyde groups, respectively) per g of CHT and CN and per g of hydrogel.

	mmol/g of Compound	µmol/g of Hydrogel
CHT	5.0	151 (3% w/w)
CN	7.6	76 (1% w/w)

**Table 4:** Linear equations and determination coefficients of the First Order, Zero Order, Higuchi, and Kosmeyer-Peppas models applied to the average data obtained from the release study under dynamic flow conditions.

CHT/PCD/CN	3:0:1	3:2:1
First Order	y = −0.0075x + 1.856	y = −0.0042x + 1.7076
	r² = 0.7547	r² = 0.4444
Zero Order	y = 1.0827x + 27.997	y = 0.4834x + 48.484
	r² = 0.6958	r² = 0.3984
Higuchi	y = 7.1598x + 17.715	y = 3.4115x + 43.195
	r² = 0.8467	r² = 0.5521
Korsmeyer-Peppas	y = 0.4633x + 1.2689	y = 0.2911x + 1.6071
	r² = 0.9572	r² = 0.9503

**Table 5:** Concentration (%w/w) of CHT, PCD, W, and LA for the preparation of the CHT/PCD/CN hydrogel.

Formulation CHT/PCD/CN		CHT	PCD	CN	W	LA
Control	3:0:0	3	0	0	96	1
	3:0:1	3	0	1	95	1
Sample	3:2:0	3	2	0	94	1
	3:2:1	3	2	1	93	1

## Data Availability

Not applicable.

## References

[1] Zhang P., Lu J., Jing Y., Tang S., Zhu D., Bi Y. (2017). Global epidemiology of diabetic foot ulceration: A systematic review and meta-analysis. Ann. Med..

[2] Everett E., Mathioudakis N. (2018). Update on management of diabetic foot ulcers. Ann. N. Y. Acad. Sci..

[3] Zhong Y., Xiao H., Seidi F., Jin Y. (2020). Natural Polymer-Based Antimicrobial Hydrogels without Synthetic Antibiotics as Wound Dressings. Biomacromolecules.

[4] Chouhan D., Dey N., Bhardwaj N., Mandal B.B. (2019). Emerging and innovative approaches for wound healing and skin regeneration: Current status and advances. Biomaterials.

[5] Chen G., Tang W., Wang X., Zhao X., Chen C., Zhu Z. (2019). Applications of Hydrogels with Special Physical Properties in Biomedicine. Polymers.

[6] Caccavo D., Cascone S., Lamberti G., Barba A.A., Larsson A. (2017). Drug Delivery From Hydrogels: A General Framework for the Release Modeling. Curr. Drug Deliv..

[7] Ji J.-Y., Ren D.-Y., Weng Y.-Z. (2022). Efficiency of Multifunctional Antibacterial Hydrogels for Chronic Wound Healing in Diabetes: A Comprehensive Review. Int. J. Nanomed..

[8] Zarrintaj P., Khodadadi Yazdi M., Youssefi Azarfam M., Zare M., Ramsey J.D., Seidi F., Reza Saeb M., Ramakrishna S., Mozafari M. (2021). Injectable Cell-Laden Hydrogels for Tissue Engineering: Recent Advances and Future Opportunities. Tissue Eng. Part A.

[9] Kaczmarek B., Sionkowska A., Gołyńska M., Polkowska I., Szponder T., Nehrbass D., Osyczka A.M. (2018). In vivo study on scaffolds based on chitosan, collagen, and hyaluronic acid with hydroxyapatite. Int. J. Biol. Macromol..

[10] Zhong Y., Seidi F., Li C., Wan Z., Jin Y., Song J., Xiao H. (2021). Antimicrobial/Biocompatible Hydrogels Dual-Reinforced by Cellulose as Ultrastretchable and Rapid Self-Healing Wound Dressing. Biomacromolecules.

[11] Zhong Y., Seidi F., Wang Y., Zheng L., Jin Y., Xiao H. (2022). Injectable chitosan hydrogels tailored with antibacterial and antioxidant dual functions for regenerative wound healing. Carbohydr. Polym..

[12] Djekic L., Martinović M., Ćirić A., Fraj J. (2020). Composite Chitosan Hydrogels as Advanced Wound Dressings with Sustained Ibuprofen Release and Suitable Application Characteristics. Pharm. Dev. Technol..

[13] Matica M.A., Aachmann F.L., Tøndervik A., Sletta H., Ostafe V. (2019). Chitosan as a Wound Dressing Starting Material: Antimicrobial Properties and Mode of Action. Int. J. Mol. Sci..

[14] Kravanja G., Primožič M., Knez Ž., Leitgeb M. (2019). Chitosan-Based (Nano)Materials for Novel Biomedical Applications. Molecules.

[15] Andreica B.-I., Cheng X., Marin L. (2020). Quaternary ammonium salts of chitosan. A critical overview on the synthesis and properties generated by quaternization. Eur. Polym. J..

[16] Hemmingsen L.M., Škalko-Basnet N., Jøraholmen M.W. (2021). The Expanded Role of Chitosan in Localized Antimicrobial Therapy. Mar. Drugs.

[17] Shao J., Yu N., Kolwijck E., Wang B., Tan K.W., Jansen J.A., Walboomers X.F., Yang F. (2017). Biological evaluation of silver nanoparticles incorporated into chitosan-based membranes. Nanomedicine.

[18] Hua Y., Ma C., Wei T., Zhang L., Shen J. (2020). Collagen/Chitosan Complexes: Preparation, Antioxidant Activity, Tyrosinase Inhibition Activity, and Melanin Synthesis. Int. J. Mol. Sci..

[19] Deng Y., Ren J., Chen G., Li G., Wu X., Wang G., Gu G., Li J. (2017). Injectable in situ cross-linking chitosan-hyaluronic acid based hydrogels for abdominal tissue regeneration. Sci. Rep..

[20] Dumitriu R.P., Profire L., Nita L.E., Dragostin O.M., Ghetu N., Pieptu D., Vasile C. (2015). Sulfadiazine—Chitosan Conjugates and Their Polyelectrolyte Complexes with Hyaluronate Destined to the Management of Burn Wounds. Materials.

[21] Loftsson T., Saokham P., Sá Couto A.R. (2019). Self-association of cyclodextrins and cyclodextrin complexes in aqueous solutions. Int. J. Pharm..

[22] Kanjickal D., Lopina S., Evancho-Chapman M.M., Schmidt S., Donovan D. (2005). Improving delivery of hydrophobic drugs from hydrogels through cyclodextrins. J. Biomed. Mater. Res. Part A.

[23] Rohner N.A., Schomisch S.J., Marks J.M., von Recum H.A. (2019). Cyclodextrin Polymer Preserves Sirolimus Activity and Local Persistence for Antifibrotic Delivery over the Time Course of Wound Healing. Mol. Pharm..

[24] Martel B., Ruffin D., Weltrowski M., Lekchiri Y., Morcellet M. (2005). Water-soluble polymers and gels from the polycondensation between cyclodextrins and poly(carboxylic acid)s: A study of the preparation parameters. J. Appl. Polym. Sci..

[25] Palomino-Durand C., Lopez M., Cazaux F., Martel B., Blanchemain N., Chai F. (2019). Influence of the Soluble–Insoluble Ratios of Cyclodextrins Polymers on the Viscoelastic Properties of Injectable Chitosan–Based Hydrogels for Biomedical Application. Polymers.

[26] Firmino D.F., Cavalcante T.T.A., Gomes G.A., Firmino N.C.S., Rosa L.D., de Carvalho M.G., Catunda F.E.A. (2018). Antibacterial and Antibiofilm Activities of Cinnamomum Sp. Essential Oil and Cinnamaldehyde: Antimicrobial Activities. Sci. World J..

[27] Flores C., Lopez M., Tabary N., Neut C., Chai F., Betbeder D., Herkt C., Cazaux F., Gaucher V., Martel B. (2017). Preparation and characterization of novel chitosan and β-cyclodextrin polymer sponges for wound dressing applications. Carbohydr. Polym..

[28] Carneiro S.B., Costa Duarte F.Í., Heimfarth L., Siqueira Quintans J.d.S., Quintans-Júnior L.J., da Veiga Júnior V.F., Neves de Lima Á.A. (2019). Cyclodextrin–Drug Inclusion Complexes: In Vivo and In Vitro Approaches. Int. J. Mol. Sci..

[29] Mogrovejo-Valdivia A., Rahmouni O., Tabary N., Maton M., Neut C., Martel B., Blanchemain N. (2019). In vitro evaluation of drug release and antibacterial activity of a silver-loaded wound dressing coated with a multilayer system. Int. J. Pharm..

[30] Hill L.E., Gomes C., Taylor T.M. (2013). Characterization of beta-cyclodextrin inclusion complexes containing essential oils (trans-cinnamaldehyde, eugenol, cinnamon bark, and clove bud extracts) for antimicrobial delivery applications. LWT Food Sci. Technol..

[31] Yildiz Z.I., Kilic M.E., Durgun E., Uyar T. (2019). Molecular Encapsulation of Cinnamaldehyde within Cyclodextrin Inclusion Complex Electrospun Nanofibers: Fast-Dissolution, Enhanced Water Solubility, High Temperature Stability, and Antibacterial Activity of Cinnamaldehyde. J. Agric. Food Chem..

[32] Sun Q., Tang P., Zhao L., Pu H., Zhai Y., Li H. (2018). Mechanism and structure studies of cinnamaldehyde/cyclodextrins inclusions by computer simulation and NMR technology. Carbohydr. Polym..

[33] Rieger K.A., Schiffman J.D. (2014). Electrospinning an essential oil: Cinnamaldehyde enhances the antimicrobial efficacy of chitosan/poly(ethylene oxide) nanofibers. Carbohydr. Polym..

[34] Zhou Z., Wang C., Bai J., Zeng Z., Yang X., Wei B., Yang Z. (2022). Cinnamaldehyde-modified chitosan hybrid nanoparticles for DOX delivering to produce synergistic anti-tumor effects. Front. Bioeng. Biotechnol..

[35] Malheiro J.F., Maillard J.-Y., Borges F., Simões M. (2019). Evaluation of cinnamaldehyde and cinnamic acid derivatives in microbial growth control. Int. Biodeterior. Biodegrad..

[36] Domadia P., Swarup S., Bhunia A., Sivaraman J., Dasgupta D. (2007). Inhibition of bacterial cell division protein FtsZ by cinnamaldehyde. Biochem. Pharmacol..

[37] Topa S.H., Subramoni S., Palombo E.A., Kingshott P., Rice S.A., Blackall L.L. (2018). Cinnamaldehyde disrupts biofilm formation and swarming motility of Pseudomonas aeruginosa. Microbiology.

[38] Xu J., Lin Q., Sheng M., Ding T., Li B., Gao Y., Tan Y. (2022). Antibiofilm Effect of Cinnamaldehyde-Chitosan Nanoparticles against the Biofilm of Staphylococcus aureus. Antibiotics.

[39] Higuchi T., Connors K.A. (1965). Advances in analytical chemistry and instrumentation. Index to Reviews, Symposia Volumes and Monographs in Organic Chemistry.

[40] Kfoury M., Landy D., Fourmentin S. (2018). Characterization of Cyclodextrin/Volatile Inclusion Complexes: A Review. Molecules.

[41] Kfoury M., Auezova L., Greige-Gerges H., Fourmentin S. (2015). Promising applications of cyclodextrins in food: Improvement of essential oils retention, controlled release and antiradical activity. Carbohydr. Polym..

[42] Korsmeyer R.W., Peppas N.A. (1981). Effect of the morphology of hydrophilic polymeric matrices on the diffusion and release of water soluble drugs. J. Membr. Sci..

[43] Higuchi T. (1963). Mechanism of Sustained-Action Medication. Theoretical Analysis of Rate of Release of Solid Drugs Dispersed in Solid Matrices. J. Pharm. Sci..

